# Immunological Changes at Point-of-Lay Increase Susceptibility to *Salmonella enterica* Serovar Enteritidis Infection in Vaccinated Chickens

**DOI:** 10.1371/journal.pone.0048195

**Published:** 2012-10-25

**Authors:** Claire E. Johnston, Catherine Hartley, Anne-Marie Salisbury, Paul Wigley

**Affiliations:** Zoonotic Infections of Pigs and Poultry Group, Institute of Infection and Global Health and School of Veterinary Science, University of Liverpool, Leahurst Campus, Neston, United Kingdom; University of California, Davis, United States of America

## Abstract

Chicken eggs are the main source of human *Salmonella enterica* serovar Enteritidis infection. *S.* Enteritidis infects the oviduct and ovary of the chicken leading to infection of developing eggs. Therefore, control in poultry production is a major public health priority. Vaccination of hens has proved successful in control strategies in United Kingdom leading to a 70% drop in human cases since introduced. However, as hens reach sexual maturity they become immunosuppressed and it has been postulated this leads to increased susceptibility to *Salmonella* infection. In this study we define the changes to the systemic and reproductive tract-associated immune system of hens throughout sexual development by flow cytometry and histology and determine changes in susceptibility to experimental *S*. Enteritidis challenge in naive and vaccinated hens. Changes to both systemic and local immune systems occur in chickens at sexual development around 140 days of age. The population of several leukocyte classes drop, with the greatest fall in CD4+ lymphocyte numbers. Within the developing reproductive tract there an organised structure of lymphocytic aggregates with γδ-T lymphocytes associated with the mucosa. At point-of-lay, this organised structure disappears and only scattered lymphocytes remain. Protection against *Salmonella* challenge is significantly reduced in vaccinated birds at point-of-lay, coinciding with the drop in CD4+ lymphocytes. Susceptibility to reproductive tract infection by *Salmonella* increased in vaccinated and naïve animals at 140 and 148 days of age. We hypothesise that the drop in γδ-T lymphocytes in the tract leads to decreased innate protection of the mucosa to infection. These findings indicate that systemic and local changes to the immune system increase the susceptibility of hens to *S*. Enteritidis infection. The loss of protective immunity in vaccinated birds demonstrates that *Salmonella* control should not rely on vaccination alone, but as part of an integrated control strategy including biosecurity and improved animal welfare.

## Introduction

*Salmonella enterica* serovar Enteritidis is the most common cause of human non-typhoidal salmonellosis in Europe and North America and is frequently associated with consumption of infected eggs. The public health and economic impact of egg infection is illustrated by the recall of over 500 million potentially contaminated eggs during an outbreak in the USA in August 2010 [1]. Egg contamination with *S*. Enteritidis is a consequence of its ability to colonise the chicken reproductive tract leading to transovarian vertical transmission to both eggs and chicks [2]–[5]. It is considered that infection of the ovary by *Salmonella* leads to yolk contamination, infection of the upper parts of the oviduct prior to the shell-gland (infundibulum and magnum) lead to contamination of the albumen or egg white and faecal contamination of the egg surface may occur after laying [6]. Tropism for the reproductive tract in *S. enterica* is largely confined to *S*. Enteritidis and the genetically similar avian-adapted serovar *S*. Pullorum and as such transovarian transmission is largely confined to these serovars [4], [5], [7]. The mechanisms that allow S. Enteritidis to colonise the avian reproductive tract are complex and multi factorial but include adhesins such as fimbriae that facilitate attachment to the reproductive mucosa [8]–[10]. Immunological changes in the host may also underlie infection of the reproductive tract and previously we have shown that suppression of cell-mediated immunity at point-of-lay in hens is a critical factor in reproductive tract infection by *S*. Pullorum [11]. Infection with *S*. Pullorum is characterised by a persistent systemic carrier state with reproductive tract infection occurring as part of a recrudescent infection at point-of-lay [7]. It has been postulated that immunosuppression in production hens may lead to reproductive tract infection with *Salmonella* carried systemically or in the gastrointestinal tract, or that *Salmonella* bacteria within the environment of the poultry house may utilise this immunosuppressive event as a ‘window of opportunity’ to infect hens.

Cost-effective control of *S*. Enteritidis is a major concern both to maintaining public health and for the poultry industry that operates on marginal costs. Within Europe, most notably the United Kingdom, changes in legislation and voluntary control schemes have led to the successful reduction in *S*. Enteritidis in eggs and as a consequence in reducing human cases from over 22000 confirmed cases in 1997 in England and Wales to fewer than 3000 by 2010 [12]. The fall in cases of *S*. Enteritidis Phage Type 4, responsible for the majority of egg-associated infection in the UK in the 1980s and 1990s, was even more marked, with a drop from over 15000 cases in 1992 to 459 in 2010. Central to the control strategy was the introduction of vaccination for laying hens in 1998. Initially vaccination was through intramuscular delivery of inactivated killed vaccines, although this has been largely superseded by the use of live attenuated vaccines that are both more effective and can be delivered through drinking water thereby reducing labor costs [13]. Vaccination has been successful to the extent that cases of salmonellosis linked to consumption of eggs produced commercially in the UK are rare. However, not all countries in Europe permit the use of live *Salmonella* vaccines, and there is considerable pressure to extend vaccine withdrawal periods prior to the point-of-lay. It is also unclear to what extent immunosuppression at point-of-lay effects protection afforded by vaccination. In the UK, the introduction of a voluntary code of practice of which vaccination was a component (Lion Mark Scheme) and subsequently legislation through the National Control Plans for *Salmonella* has also led to considerable improvements in biosecurity, hygiene and husbandry practice in the poultry industry that complement vaccination and as such it is impossible to determine whether vaccination alone would have had such an impact on the control of *Salmonella* in eggs.

In this study we aimed to determine the systemic and local changes in the reproductive tract immune system that underlie immunosuppression at point-of-lay and whether these have an effect on susceptibility to *S*. Enteritidis infection in naive or vaccinated chickens. Relatively little is known about the immunological structure and particularly adaptive responses in the healthy or infected avian reproductive tract other than basic descriptive histology. We set out to investigate the changes in cell populations and structure and cytokine repertoire expressed in the hen ovary and oviduct, along with changes in the circulating T lymphocyte populations. We then determined whether these changes correspond to increased susceptibility to *Salmonella* infection. Understanding susceptibility to *Salmonella* and any deficiency in vaccine protection at point-of-lay will allow development of more effective vaccination and other control strategies, reducing the risk of *S*. Enteritidis infection and subsequent transmission to eggs.

## Materials and Methods

### Ethics Statement

All work was conducted in accordance with UK legislation governing experimental animals under project licence PPL 40/3063 and was approved by the University of Liverpool ethical review process prior to the award of the licence. All animals were checked a minimum of twice daily to ensure their health and welfare.

### Experimental animals

1-day old female Lohmann Brown Egg laying Chicks were obtained from a commercial hatchery. Birds were vaccinated against Marek's Disease Virus in the hatchery, but not against any other pathogen. Chicks were maintained in floor pens and given *ad libitum* access to water and a vegetable protein based diet (SDS, Witham, Essex UK). Chicks were maintained at a temperature of 30°C until 21 days of age then lowered to 20°C.

### Determining Immunological changes at point-of-lay

45 chicks were housed as described above. At 32, 60, 77, 102, 124, 132, 140, 148 and 165 days of age, five birds were killed by neck dislocation and immediately dissected. Spleens were removed and processed individually for flow cytometry and histology as described below. At 102 days of age onwards, ovarian tissue and tissue from three regions of the oviduct: isthmus, magnum and uterus were taken for histology or frozen in RNAlater (Life Technologies, Paisley, UK) for analysis of cytokine expression. Breast muscle was taken as a control tissue for comparison.

### Analysis of changes to systemic immune system by flow cytometry

Splenic tissue taken from individual birds at post mortem examination was placed into 5 ml Dulbecco's Modified Essential Media containing 5% foetal calf serum (Life Technologies, Paisley, UK). Cell suspensions were prepared by passing tissue through a 40 μM cell strainer (Beckton Dickinson, Oxford, UK,). The suspension was overlaid onto 10 ml room temperature Histopaque 1083 and spun at 220×*g* for 10 minutes. The buffy coat layer was then removed and resupended into FACS Buffer (PBS with 1% BSA and 0.005% sodium azide). Cells were counted in a haemocytometer and adjusted to 10^6^ cells/ml prior to staining.

For antibody staining, 10^6^ cells in a volume of 1 ml were pelleted in a microcentruge for 3 ml. Supernatant was removed and 2 μl of primary antibody added and the pellet resuspended in residual buffer. The following antibodies were used: mouse anti-chicken CD4, CD8α, CD8β, MHC Class II, TCRγδ, the B lymphocyte marker Bu-1 and the monocyte-macrophage marker KUL01 (Southern Biotech, Birmingham, Alabama, USA) along with an IgG1 isotype control, clone MOPC 21 (Sigma, Poole, UK) as an isotype control. Cells were then incubated at room temperature for 10 minutes. 1 ml of FACS buffer was added, the tube inverted to mix and cells pelleted in a microcentrifuge and supernatant removed. This step was repeated to wash the primary antibody. After washing, 2 μl of FITC- conjugated rabbit anti-mouse IgG (AbD Serotec Cambridge, UK) was added and incubated in the dark at room temperature for 10 minutes. The cells were then washed as described above and resuspended in 600 μl of FACS buffer containing 1% paraformaldehyde and then stored at 4°C until analysed. Cells were analysed on a Becton Dickinson FACScalibur flow cytometer. Analysis of cells populations was performed using CellQuestPro (Beckton Dickinson) and WinMDI 2.8 (www.http://facs.scrips.edu/software.html) software.

### Immunhistochemistry

Tissue samples were embedded in OCT, snap frozen, cut into 8 μm sections on a cryostat and stained with mouse anti-chicken monoclonal antibodies (Southern Biotech,) to CD4, CD8α, CD8β, γδ T cell receptor, MHC Class II and the macrophage marker KUL01. Antibody concentrations and incubation conditions were based on those described by WIthanage et al [14], [15]. Visualisation was determined by use of the Vectastain Elite ABC kit containing biotinylated anti-mouse IgG, avidin and biotinylated horseradish peroxidase. The complex was then visualised with the NovaRED substrate kit (Vector Labs, Peterborough, Cambs, UK). Sections were examined and photographed using a NIKON Eclipse 80i microscope. Analysis of cell populations was determined by the methods described by WIthanage et al [15], [16].

### Cytokine expression in the reproductive tract

Expression of the key cytokines IL-2, IL-4, IL-6, IL-12, IFN-γ and the chemokines CXCLi2 and CXCLi4 were determined in oviduct and ovarian tissue by qRT-PCR as described previously [7], [15]. Breast muscle tissue was taken as a negative control as it considered to have no cytokine expression. RNA was isolated from tissue samples using RNeasy mini kits (Qiagen, Crawley, Sussex. UK). Samples stored in RNAlater were removed and placed into the lysis buffer provided and homogenised using a TissueLyser (Qiagen). RNA was then isolated according to manufacturer's instructions. RNA levels between samples was normalised based on expression on 28S rRNA and expression data presented as a 40-Ct value minus the mean Ct value for breast muscle [7].

### *S*. Enteritidis challenge in vaccinated and naive animals

52 one-day old commercial female egg-laying chicks were housed in two equal sized groups under the conditions described above. One group were vaccinated at 8, 41 and 95 days of age with a commercial live vaccine (Avipro SE, Lohmann Animal Health, Cuxhaven, Germany) according to manufacturer's instructions. At 77, 102, 132, 140 and 148 days of age 5 chickens from each group were housed separately and infected intravenously via the wing vein with 10^6^ cfu of *S*. Enteritidis 125109 PT4 [4], [17] in a volume of 0.2 ml Luria Bertani Broth. At four days post challenge, birds were killed by neck dislocation. At post mortem examination spleen and liver samples were aseptically collected for quantitative bacteriology with counts determined on Brilliant Green Agar (Oxoid) using previously described methods [17]. The reproductive tract was removed and ovary and oviduct tissue samples from upper (infundibulum) and mid (magnum) of the oviduct were collected and pooled from each individual bird, incubated overnight with Selenite Broth and then plated onto Brilliant Green Agar to detect presence or absence of *Salmonella* [17]. Developing eggs in the oviduct or ones laid by birds after challenge were collected into jars containing Selenite Broth for detection of *Salmonella* as previously described [18].

### Statistical analysis

Statistical analysis was performed using SPSS v.20 (IBM). Comparison between cell populations was made by ANOVA. For comparison of bacterial load between infected groups through the Kruskal-Wallis test, an equivalent non-parametric test to ANOVA, was used as the data was not distributed normally.

## Results

### Changes in leucocyte populations at point-of-lay

**Figure 1:**
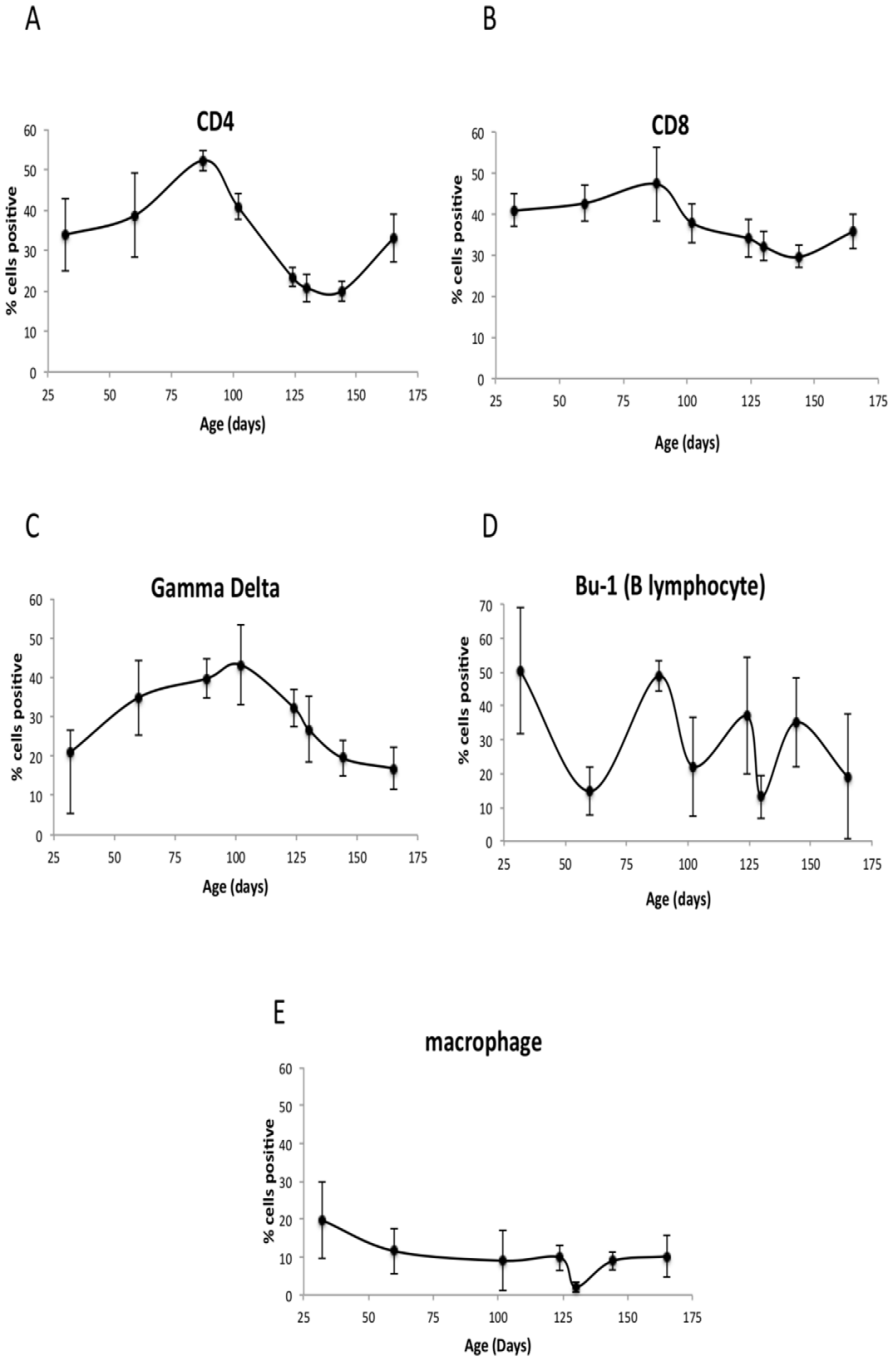
Changes in splenic mononuclear leucocyte populations through development of and onset of sexual maturity in commercial laying hens determined by flow cytometry following specific single color staining with monoclonal antibodies to major chicken cell markers. Fig. 1A shows the CD4+ population which falls significantly between 102 and 130 days of age as does the γδ population (Figure 1C). Figure 1B shows the CD8+ population (data for CD8α and CD8β combined), Figure 1D shows B lymphocyte (Bu-1) and Figure 1E macrophage (KUL01) stained populations. Data are shown as means of the percentage of positive cells (± SD). Data shown are based on five birds per time point.

As birds move towards sexual maturity at 130 to 140 days of age there is a decline in T lymphocyte populations in the spleen (Fig. 1a, 1b, 1c, whereas there is no consistent change in macrophage or B lymphocyte populations (Figure 1d, 1e). Comparison of the population at 88 or 102 days of age to that at 130 days of age shows a significant falls in both CD4+ (Fig. 1a) and γδ T cell (Fig. 1b) populations (*P* = <0.001). CD8+ populations (CD8α and CD8β combined) (Fig. 1c) also show a decline between 102 and 130 days of age, though this is not statistically significant (*P* = 0.062). The decline in these lymphocyte populations matches the start of the egg-laying period (typically 125–140 days of age in commercial layers). At 165 days of age when hens are fully productive there is a significant recovery in CD4+ populations from the levels at 130 days (*P* = 0.03). However, γδ levels remain low as birds mature. The B lymphocyte population was considerably variable from bird-to-bird and between time points though there was no discernable change in population.

### Cellular populations and changes in the chicken reproductive tract

**Figure 2:**
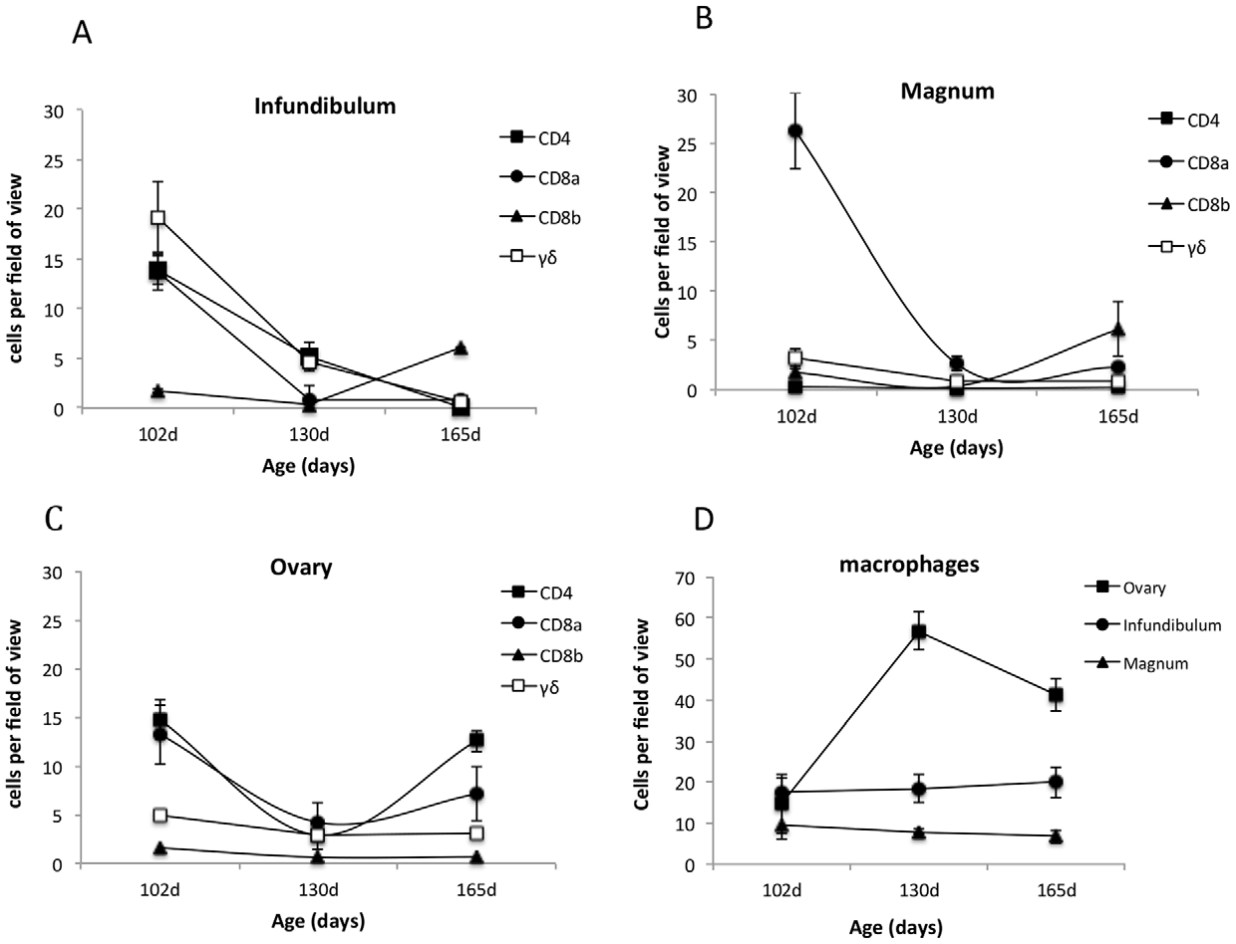
Changes in cell populations in the chicken reproductive tract as determined by immunohistological examination. 2A shows T lymphocyte populations in the infundibulum (upper oviduct), 2B in the magnum (mid oviduct) and 2Cthe ovary. Figure 2D shows the changes in macrophage populations in the ovary and oviduct. Data are based on 10 fields of view for two sections of each tissue from each animal. Between five to seven birds were examined at each time point. Data are given as a mean (±SD).

**Figure 3:**
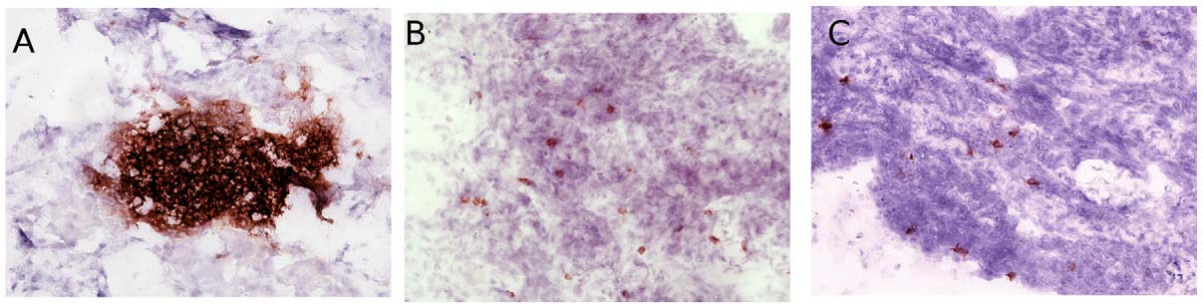
Changes to CD4+ lymphocyte populations in the reproductive tract. At 102 days of age CD4+ populations are primarily organised into aggregates in the ovary (Figure 3A) and oviduct. Birds lack lymph nodes and form more loose lymphoid structures. At 130 days (Figure 3B) these structures are no longer found, with smaller populations of cells found throughout the ovary. At 165 days (Figure 3C) CD4+ numbers increase slightly, but remain scattered throughout the tissue. Photomicrographs taken at a magnification of ×400.

Leucocyte populations in the reproductive tract show considerable changes in numbers and organisation through the onset of sexual maturity (Figure 2). In the developing oviduct of the immature hen at 105 days of age, T lymphocytes are organised into aggregates and scattered in small numbers throughout the infundibulum (upper) (Figure 2a) and magnum (central) oviduct (Figure 2b), although there are more CD4+ cells in the infundibulum and CD8a+ cells in the magnum. Both are numerous in ovarian tissue (Fig. 2c). As birds approach point-of-lay at 130 days of age, lymphoid aggregates disappear with lower overall numbers of lymphocytes scattered through the reproductive tract (Figure 3). Significantly, there is a large population of γδ T lymphocytes in the infundibulum of the developing oviduct that declines at sexual maturity. Although numbers are fewer in the magnum and ovary, a similar decline is seen. By 165 days of age there is an increase in lymphocyte numbers from the levels at 130 days of age throughout the reproductive tract with the ovary showing the greatest increase in lymphocyte numbers. The exception being γδ T lymphocytes which remain at low levels. In contrast, there is little change in macrophage populations in the oviduct throughout sexual development, although there is an increase in macrophages in ovary towards at point-of-lay (Fig. 2d). Staining for MHC Class II that are neither stained by macrophage or B lymphocyte markers also reveals an organised structure of cells below the epithelium that may represent an organised population of antigen presenting cells in the reproductive that have not previously described.

### Expression of cytokines in the reproductive tract

**Figure 4:**
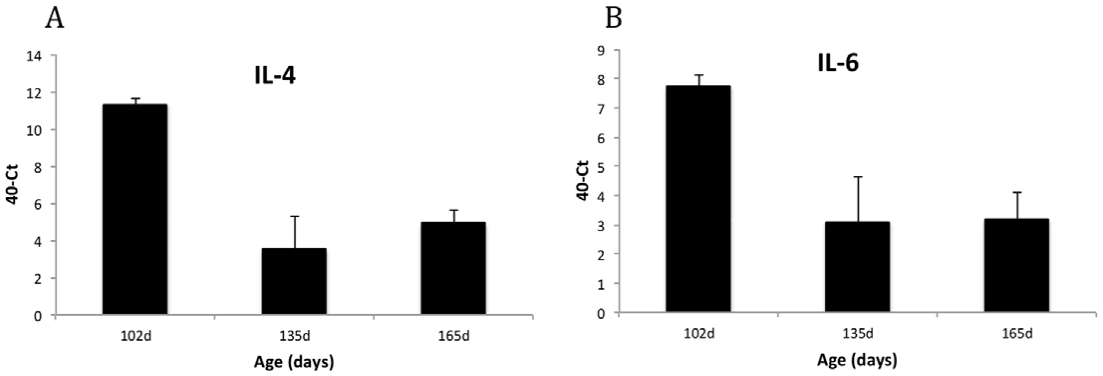
Expression of Interleukin-4 (A) and Interleukin-6 (B) in the ovary. Data is expressed as 40-Ct values in ovarian tissue in comparison to expression in breast muscle tissue as a control for no expression (40-Ct = 0). RNA levels are normalised between tissues using 28S RNA expression. Data are shown as means based on expression levels of 5 birds at each time point (±SD).

Expression of IL-4 and some expression of IL-6 were detected in the ovaries at all time points examined (Fig. 4). Similar levels expression of IL-6 was found in both the infundibulum and magnum within the oviduct, though expression of IL-4 was detectable throughout the oviduct, levels were lower than the ovaries (data nor shown). Given that secretion of IgA has been shown in the ovary and oviduct, IL-4 may be playing a role in regulation of antibody secretion. No expression of other cytokines or chemokines could be detected.

### Susceptibility to *S*. Enteritidis infection

*Salmonella* levels in the spleen, liver and reproductive tract following challenge in vaccinated and naive chickens are shown in Table 1. Prior to sexual maturity vaccinated birds showed both lower levels of *Salmonella* and fewer positive animals compared to naive animals. Significantly lower bacterial numbers were found in the spleen of vaccinated chickens challenged at 77 (*P* = 0.048) and 102 days of age (*P* = 0.009) than the naïve group indicating a significant level of protection. However protection decreased as birds reached point-of-lay. *Salmonella* levels in the spleen were not lowered in the vaccinated group in birds challenged at 132 (*P* = 0.75), 140 (*P* = 0.33) 0r 148 days of age (*P* = 0.61). Reproductive tract infection also coincides with the reaching of sexual maturity in vaccinated birds with the ovaries or oviduct only being infected following challenge at 140 and 148 days of age. There also appeared to be an increased susceptibility to reproductive tract infection in naïve animals at point-of-lay with three out of five birds positive at 148 days of age. Developing and laid eggs from both groups post-challenge were found to contain *Salmonella* with 3 of 21 eggs laid by naïve hens and 1 of 14 eggs laid by vaccinated hens cultured positive for the presence of *Salmonella*.

**Table 1:** *Salmonella* Enteritidis infection levels in spleen, liver and reproductive tract following challenge of vaccinated and naïve hens four days following intravenous challenge at varying ages through sexual development.

Group	Tissue	Age at challenge (days)									
		77d		102d		132d		140d		148d	
		No positive/total number	Median log_10_ cfu/g (range)	No positive/total number	Median log_10_ cfu/g (range)	No positive/total number	Median log_10_ cfu/g (range)	No positive/total number	Median log_10_ cfu/g (range)	No positive/total number	Median log_10_ cfu/g (range)
**Vaccinated**	Spleen	2/5	0.85 (0–3.0)	2/5	1.09 (0–2.18)	5/5	1.85 (1.2–2.48)	4/5	1.0 (0–2.18)	1/5*	<1
	Liver	0/5	<1	1/5	0.58 (0–1.70)	2/5*	<1	3/5	1.08 (0–2.90)	2/5*	<1
	Ovary & Oviduct	0/5	ND	0/5	ND	0/5	ND	2/5	ND	2/5	ND
**Naive**	Spleen	3/5	2.6 (0–2.18)	5/5	2.49 (1.7–3.48)	3/5	2.57 (0–4.18)	3/5	1.33 (0–2.70)	3/5	0.68 (0–2.74)
	Liver	1/5	0.58 (0–1.70)	2/5	1.15 (0–2.80)	3/5	0.66 (0–3.30)	3/5	0.56 (0–2.8)	3/5	1.15 (0–2.90)
	Ovary & Oviduct	0/5	ND	1/5	ND	1/5	ND	2/5	ND	3/5	ND

Data are presented as median values with ranges based on five birds per group at each age and number positive for *Salmonella* out of total number challenged per group. Quantitation was not performed on pooled reproductive tract samples and are shown as *Salmonella* culture positive or negative following enrichment culture.

* Positive only after enrichment.

ND = Not done.

## Discussion

The data presented here show that susceptibility to *S*. Enteritidis infection in the chickens is increased around the point-of-lay and that the efficacy of vaccination is significantly decreased at this time, although vaccination offers protection prior to sexual maturity. Moreover, challenge at point-of-lay can lead to infection of the reproductive tract and eggs even in vaccinated birds. Previously we have shown that suppression of T lymphocyte activity was associated with infection of the reproductive tract by *Salmonella* [19]. Here we show that a sharp decrease in T lymphocytes and particular CD4+ cells underlies this loss of activity. Furthermore there are substantial changes to the local organisation of lymphocytes within the reproductive tract and, in particular, a sharp drop in γδ T lymphocytes. These changes are likely to increase the susceptibility of the ovarian and oviductal epithelium to infection.

Hens at point-of-lay have increased susceptibility for infection with *Salmonella* even following vaccination and this has potential consequences for its control. Whilst the relatively high challenge inoculum delivered by the intravenous route used in these experiments is more likely to lead to reproductive tract infection or egg infections than a low-level challenge in the field, the model used clearly shows that vaccinated hens are more susceptible to *Salmonella* infection at point-of-lay. There is good epidemiological evidence from the UK and other countries that vaccination is effective in reducing egg infection [20], however the data presented here would indicate that protection afforded by vaccination is not complete. It is perhaps significant to consider that vaccination in the UK has also been accompanied by improvements in hygiene, biosecurity and animal welfare within the poultry industry [21]–[23]. This has reduced levels of contamination in poultry houses and considerably reduced the likelihood of exposure to *Salmonella* infection. It does, however, suggest that both vaccination and improved biosecurity and husbandry should be employed in concert to control salmonellosis in egg production and that vaccination alone may not offer complete protection.

The decrease in T lymphocyte proliferation activity at point-of-lay previously described appears to be related to or coincide with a reduction in cell numbers. There is a reduction in several T cell subsets at point-of-lay indicating that immunosuppression is not liked to changes in a single cell type. However the most marked reductions are in CD4+ and γδ cells that is likely to lead to reduced production of Th1 cytokines such as interferon-γ that drive the cellular responses associated strongly with clearance and protection against salmonellosis in the chicken [7], [15], [24]. It is probable that these changes are mediated by changes in steroid hormones that drive development of the reproductive tract and the onset of oviposition and could be considered analogous to immunosuppression during pregnancy or post-partuition in mammalian species, although the increase in nutritional requirements that underlies rapid development of the reproductive tract and oviposition may also have effects upon the immune system [25]. The role of local immunological changes in the chicken reproductive tract on infection is less clear. In the developing oviduct and ovary there is an organised structure of lymphocytes in aggregates that disperse at point-of-lay. Birds, unlike mammals, lack encapsulated lymph nodes instead developing less structured lymphoid aggregates particularly upon antigenic stimulation of the immune system [26]. Previously, lymphoid structures such as those containing CD4+ cells described here had been found in the oviduct of immature and older hens, although their cellular composition was not described [27], [28]. The dispersion of these lymphocyte aggregates at point-of-lay has not been described previously, nor has the relatively high numbers of γδ T lymphocytes within the reproductive tract. Although there is some drop in T cell numbers overall in the reproductive tract at point-of-lay, CD4, CD8α, CD8β and γδ T cells remain scattered throughout the oviduct and ovary throughout the onset of lay. The drop in γδ T lymphocytes may be significant in the increased susceptibility to reproductive tract infection, particularly as both vaccinated and non-vaccinated birds show increased infection levels. Whilst it is considered that γδ T lymphocytes play an important role in innate defences in mucosal tissues including the mammalian reproductive system, such function in the avian reproductive tract has not yet been described. Furthermore it is known that circulating γδ T lymphocytes play an important role in the initiation of immune responses to *S*. Enteritidis in the chicken [29], so a decrease in these cells in the ovary and oviduct may have consequences in reproductive tract infection. In contrast, there is little change in macrophage numbers in the oviduct whereas there is an influx of macrophages or macrophage-like cells into the ovaries, as previously described [30]. However neither the function nor the phenotype of these cells is known.

The relatively scattered nature and small numbers of leucocytes in the reproductive tract are perhaps reflected in the lack of detectable expression of cytokines in the reproductive tract, with the obvious exception of IL-4. Previously the presence of IgA, IgM and IgG secreting B cells and secreted antibodies have been shown in the oviduct and ovary and, as such, the expression of IL-4 in these tissues is not surprising and fits well with the known humoral immune responses of the ovaries and oviduct [28].

Although susceptibility to infection of the reproductive tract increases in both naïve and vaccinated birds increases at point-of-lay, susceptibility to splenic infection is only increased in vaccinated birds. The drop in T lymphocytes in the spleen is a potential mechanism for this. Cellular immunity is key to both clearance of primary infection and to protection to systemic re-challenge with *Salmonella* [24]. The significant reduction in T lymphocyte numbers in the spleen could potentially lead to a failure to mount an effective rapid anamnestic response in vaccinates leading to increased susceptibility to systemic infection leading to increased the increased bacterial numbers in the spleen found. The changes in T lymphocytes populations appear to have no impact on susceptibility in naïve animals. However, in the *Salmonella* challenge model used it is likely that there would be a limited adaptive response within four days of primary challenge and so little difference in susceptibility if mediated by cellular immunity. It is possible that subsequent clearance of infection during the period of immunosuppression is slower. Although the data presented here and previously show that there is both a decrease in protection in vaccinated hens and increased susceptibility to recrudescent *Salmonella* infection in carrier hens [31], the effect of the drop of T lymphocyte numbers on a primary systemic *Salmonella* infection requires further investigation, though susceptibility to reproductive tract infection does appear to increase.

Data from the field and experimental observations clearly indicate that vaccination is an invaluable tool in the control of infection of table eggs with *Salmonella* Enteritidis [20], [32]. However, the immunological changes that accompany sexual maturity and the onset of egg-laying in hens mean that at this point there is increased susceptibility to infection in both naive and vaccinated animals. It may be possible to improve vaccines and a number of promising live attenuated and sub-unit vaccine candidates have been trialled experimentally [33]–[38] as have attempts to stimulate immunity to *S*. Enteritidis through cytokines and Toll-like receptor agonists [39]–[42]. However, it is not clear if these approaches would overcome any gap in immunity and issues of cost along with regulatory issues on the use of genetically modified bacterial vaccines in many countries may limit their use. As such it would be prudent for the poultry industry worldwide to adopt a holistic approach to *Salmonella* control that combines vaccination with other measures such as good husbandry, biosecurity and welfare.
